# Urine proteomics in the diagnosis of stable angina

**DOI:** 10.1186/s12872-016-0246-y

**Published:** 2016-04-19

**Authors:** Ulf Neisius, Thomas Koeck, Harald Mischak, Sabrina H. Rossi, Erin Olson, David M. Carty, Jane A. Dymott, Anna F. Dominiczak, Colin Berry, Keith G. Oldroyd, Christian Delles

**Affiliations:** BHF Glasgow Cardiovascular Research Centre, Institute of Cardiovascular and Medical Sciences, University of Glasgow, 126 University Place, Glasgow, G12 8TA UK; mosaiques diagnostics GmbH, Rotenburger Str. 20, 30659 Hannover, Germany; Golden Jubilee National Hospital, Agamemnon Street, Clydebank, G81 4DY UK

**Keywords:** Coronary artery disease, Urinary proteomics, Stable angina

## Abstract

**Background:**

We have previously described a panel of 238 urinary polypeptides specific for established severe coronary artery disease (CAD). Here we studied this polypeptide panel in patients with a wider range of CAD severity.

**Methods:**

We recruited 60 patients who underwent elective coronary angiography for investigation of stable angina. Patients were selected for either having angiographic evidence of CAD or not (NCA) following coronary angiography (*n* = 30/30; age, 55 ± 6 vs. 56 ± 7 years, *P* = 0.539) to cover the extremes of the CAD spectrum. A further 66 patients with severe CAD (age, 64 ± 9 years) prior to surgical coronary revascularization were added for correlation studies. The Gensini score was calculated from coronary angiograms as a measure of CAD severity. Urinary proteomic analyses were performed using capillary electrophoresis coupled online to micro time-of-flight mass spectrometry. The urinary polypeptide pattern was classified using a predefined algorithm and resulting in the CAD_238_ score, which expresses the pattern quantitatively.

**Results:**

In the whole cohort of patients with CAD (Gensini score 60 [40; 98]) we found a close correlation between Gensini scores and CAD_238_ (*ρ* = 0.465, *P* < 0.001). After adjustment for age (*β* = 0.144; *P* = 0.135) the CAD_238_ score remained a significant predictor of the Gensini score (*β* =0.418; *P* < 0.001). In those with less severe CAD (Gensini score 40 [25; 61]), however, we could not detect a difference in CAD_238_ compared to patients with NCA (−0.487 ± 0.341 vs. −0.612 ± 0.269, *P* = 0.119).

**Conclusions:**

In conclusion the urinary polypeptide CAD_238_ score is associated with CAD burden and has potential as a new cardiovascular biomarker.

**Electronic supplementary material:**

The online version of this article (doi:10.1186/s12872-016-0246-y) contains supplementary material, which is available to authorized users.

## Background

Stable angina, with an estimated prevalence of 2–4 % in most European countries [1], is a common disorder. Contemporary guidelines recommend assessment of patients with suspected angina with a variety of non-invasive tests for diagnostic purposes [1, 2]. For reasons of availability, cost and test performance there is a need for new diagnostic tests to contribute to the diagnostic process of stable angina.

Recent advances in proteomics have allowed studies into new biomarkers of cardiovascular disease [3]. A variety of biofluids can be subject to proteomic analysis, yet for clinical applications urine has several advantages over blood [4]. Polypeptide expression patterns in urine can represent a “peptide finger print” of a disease and can therefore function as a disease specific biomarker.

We have previously developed a urinary proteome signature specific for coronary artery disease (CAD). An initial small study characterized a 15-biomarker panel with sensitivity and specificity to predict the presence of CAD of 98 and 83 %, respectively [5]. In our most recent study in a total of 623 individuals, a 238-biomarker panel in patients and controls from different centers was validated and filtered against concomitant disease and treatment-specific effects. The panel had a sensitivity of 79 % and a specificity of 88 % for the diagnosis of CAD [6]. The majority of CAD patients in this cohort had severe CAD and was scheduled for surgical revascularization.

Here, in a scenario closer to potential clinical use, we investigated whether the 238-biomarker panel (CAD_238_) could also differentiate patients with less extensive CAD from patients without angiographic evidence of CAD (NCA).

## Methods

### Study end points

The primary end point of this study was the differentiation of significant CAD from NCA by the CAD_238_ score in patients with stable angina like chest pain. Secondary end points included: CAD_238_ score correlation with CAD extent/severity; evaluation of potential co-founding factors and assessment of the CAD_238_ scores capacity to identify prognostic relevant CAD.

### Patients

Study participants were referred by local cardiologists for further investigations of typical symptoms and/or positive non-invasive tests to the Golden Jubilee National Hospital, Clydebank, UK, and underwent elective coronary angiography in 2009. From all patients (*n* = 1380) we selected those with significant CAD, defined as at least one coronary artery stenosis ≥75 % of the artery lumen; or NCA, defined as the absence of any angiographic evidence of CAD. Study visits took place in 2010, 286 (58 to 459) days after the coronary angiography/percutaneous intervention, and included urine sample collection. Patients were excluded for the following reasons: history of established CAD, acute coronary syndromes, previous organ transplantation, heart failure, malignant concomitant diseases within the last 5 years, systemic inflammatory diseases or severe liver diseases. Based on medical record information, residence within 13 miles of the study centre and availability of contact information, 260 eligible patients were invited of whom 87 agreed to participate (Additional file 1: Figure S1). Six patients had to be excluded following assessment at the study visit. Of the remaining participants, we selected a cohort of 30 patients with CAD and 30 age-matched patients with NCA for proteomic analysis.

Further cases and controls from a previous study [6] were included in the quantitative analyses in the present study in order to represent the spectrum of CAD from NCA to severe CAD requiring surgical revascularization. The cases consisted of patients with severe coronary artery disease confirmed by coronary angiography (*n* = 66) who were recruited at the pre-assessment clinic on the day prior to elective coronary artery bypass grafting (CABG). The controls were healthy volunteers without evidence of CAD (*n* = 67).

All participants gave written informed consent. The study was approved by the West of Scotland Research Ethics Committee and is in keeping with the principles of the Declaration of Helsinki.

### Gensini score

To quantify the overall CAD extent the scoring system suggested by Gensini [7] was used. Stenoses of less than 25, 25–49, 50–74, 75–94, 95–99 and 100 % were equated with 1, 2, 4, 8, 16 and 32, accordingly. Depending on the artery segment and dominance of the left or right coronary artery, these scores were multiplied with factors from 0.5 to 5 [7] to implement the functional significance of the area supplied by that segment. The artery segment scores were finally added to obtain the Gensini score. If not stated otherwise, reported Gensini scores are based on angiographic findings prior to percutaneous intervention.

### Carotid intima media thickness

Measurement of carotid intima media thickness was performed by ultrasonography (Acuson Sequoia C512, Siemens, Erlangen, Germany) with an 8 MHz linear-array transducer. The carotid intima-media thickness of the left and right common carotid was measured in the far wall, 1 cm proximal of the carotid bulb in a plaque free region in accordance with the Mannheim consensus [8]. ECG signals were stored simultaneously to define the systolic and diastolic phase of the cardiac cycle. Offline measurements were performed semi-automatically at end diastole on B-mode images using Image-Pro Plus software, version 3.0 (Media Cybernetics, Bethesda, USA). Reported carotid intima-media thickness represents the mean of all taken measurements.

### Sample collection

Routine urine and blood samples were collected according to local standards and analyzed in laboratories at Gartnavel General Hospital, Glasgow, UK. Urine samples for proteomics were stored at −80 °C at the BHF Glasgow Cardiovascular Research Centre. Proteome analysis was carried out at Mosaiques Diagnostics, Hannover, Germany.

### Urinary proteomics

All samples were prepared by ultrafiltration, desalting and lyophilisation, as described in detail previously [9]. Prior to CE-MS analysis, samples were resuspended with high performance liquid chromatography grade water to yield 0.8 g/L protein concentration as measured by bicinchoninic acid assay (Interchim, Montlucon, France).

CE-MS analysis was performed as previously described [10] using a P/ACE MDQ capillary electrophoresis system (Beckman Coulter) online coupled to a time-of-flight mass spectrometer (micro-time- of-flight MS; Bruker Daltonic). Spectra were accumulated every 3 s over a range of mass-to-charge ratios from 350 to 3000. Accuracy, precision, selectivity, sensitivity, reproducibility, and stability using this technique are described in detail elsewhere [9]. In brief, the detection limit is in the range of 1 fmol, depending on the ionization properties of the individual peptide. In a urine sample, the detection limit in the crude sample before processing is 100 to 1000 fmol/mL. Platform validation was performed as described previously [11].

MosaiquesVisu (mosaiques diagnostics and therapeutics AG, Hannover, Germany) was employed for mass spectrometry data processing. Data were normalized based on reference signals from 29 abundant “housekeeping” peptides generally present in urine, which are the result of normal biological processes and are not affected by age, sex, or disease state [12]. The individual sample data were calibrated with a local regression algorithm and 29 internal standard peptides, all collagen fragments [12], as reference. After normalization signal intensity was employed as a marker for relative quantity.

### Statistics

A power calculation (1-*β* = 0.95, *α* = 0.05) was conducted to evaluate the number of subjects necessary to detect a significant CAD_238_ score difference. Based on our previous data [6] (Additional file 1: Table S1) 12 patients in each group were required for the actual power 0.96. To account for any analytical difficulties we opted for a convenience sample of 30 patients per group.

Data were analyzed using SPSS software, version 15.0 (SPSS Inc., Chicago, USA). The CAD_238_ score corresponding to a CAD specific urinary polypeptide pattern was calculated as published by Delles et al. [6]. Normality of data distribution for all experiments was tested using the Kolmogorov-Smirnov test and visual inspection of Q-Q plots. Correlations were assessed by calculating Pearson’s or Spearman’s correlation coefficient for parametric and non-parametric data, respectively. The two sample Student’s *t* test or the Mann Whitney test was conducted as appropriate for the comparison of two groups of paired observations for continuous data. For comparison of categorical data of independent groups the Chi-squared test was employed.

## Results

**Fig. 1:**
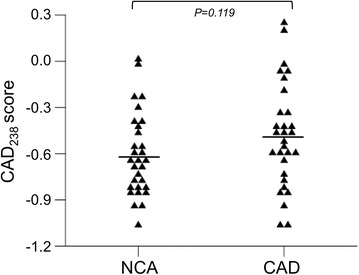
CAD_238_ score comparison between patients with NCA and CAD. *Lines* represent the mean. *NCA* normal coronary arteries, *CAD* coronary artery disease

Cardiovascular risk factors were similar in patients with or without angiographic evidence of CAD (Table 1). Similarly, in patients with angina like chest pain the CAD_238_ score was not significantly different between patients with CAD and those with NCA (−0.487 ± 0.341 vs. −0.612 ± 0.269, *P* = 0.119) as shown in Fig. 1. To adjust for potential cofounding factors we used a stepwise linear regression model with CAD_238_ score, age, gender and diabetes status as predictors of CAD. The resulting model contained only the CAD_238_ score (*β* = 0.206; *P* = 0.090) and gender (*β* = 0.128; *P* = 0.092), but remained statistically non-significant (*P* = 0.072).

**Table 1:** Cohort characteristics of stable angina patients with CAD and NCA

	CAD, *n* = 30	NCA, *n* = 30	*P*-value
CAD_238_ score	−0.487 ± 0.341	−0.612 ± 0.269	0.119
ACR (all > detection limit)	0.9 [0.6; 2.0]	1.6 [1.0; 3.4]	0.036
Age, years	55.1 ± 6.0	56.1 ± 7.0	0.539
Sex, m/f	16/14	10/20	0.192
BMI, kg/m2	27.9 ± 4.2	28.8 ± 7.5	0.605
SBP, mmHg	138 ± 17	138 ± 19	0.976
DBP, mmHg	78 ± 10	81 ± 9	0.205
Heart rate,/min	57 ± 9	59 ± 9	0.321
Total cholesterol, mmol/l	4.3 [3.8; 5.6]	4.8 [4.3; 5.7]	0.077
LDL-cholesterol, mmol/l	2.1 [1.8; 3.3]	2.4 [2.0; 3.4]	0.533
HDL-cholesterol, mmol/l	1.2 [0.9; 1.4]	1.3 [1.0; 1.6]	0.286
Triglycerides, mmol/l	1.6 [1.1; 2.1]	1.7 [1.1; 2.5]	0.404
Hypertension history, %	77	57	0.438
CAD family history, %	73	70	1.000
Diabetes history, %	20	13	0.299
Active smoking, %	23	17	0.747
Statin, %	87	57	0.020
Aspirin, %	93	33	<0.001
Beta-blocker, %	83	27	<0.001
ACEI/ARB, %	40	33	0.789
Gensini-score	40 [25; 61]	0	–
Corrected Gensini-score ‡	8 [4; 40]	0	
Carotid IMT, mm	0.724 [0.677; 0.802] ^a^	0.753 ± 0.092 #	0.444

Data are given as mean ± SD or median [ICR] as appropriate. *P*-values are from Student’s *t*-test, Mann–Whitney *U*-test, Chi-square test or Fisher’s exact test where appropriate. *ACEI* angiotensin-converting enzyme inhibitor, *ACR* albumin-creatinine ratio, *ARB* angiotensin receptor blocker, *BMI* body mass index, *CAD* coronary artery disease, *DBP* diastolic blood pressure, *IMT* intima-media thickness, *NCA* normal coronary arteries, *HDL* high-density lipoprotein, *LDL* low-density lipoprotein, *SBP* systolic blood pressure, ‡ Gensini-score after percutaneous intervention; ^a^ *n* = 29; # *n* = 28

In order to increase the sample size of our study and to cover a wider spectrum of severity of CAD for correlation analyses we added data from patients with severe CAD who were due to undergo CABG surgery (Additional file 1: Table S1). The complete CAD cohort (*n* = 96 patients) is summarized in Additional file 1: Table S2.

**Fig. 2:**
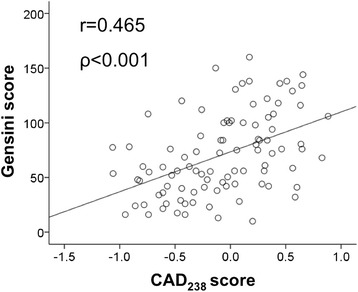
Correlation between CAD_238_ score and the Gensini score. The Gensini score (y-axis) is plotted against the CAD_238_ score (x-axis) for 96 patients. Shown are the Spearman’s correlation coefficient and the corresponding *P*-value

The Gensini score was different in patients with stable angina and CAD compared to CAD patients undergoing CABG (40 [25; 61] vs. 77 [56; 109]; *P* < 0.001). When all patients with CAD were combined (*n* = 96) the CAD_238_ score correlated closely with the Gensini score (*ρ* = 0.465, *P* < 0.001; Fig. 2). Due to the difference in age between patients with CAD and stable angina and those prior to surgical coronary revascularization (55.1 ± 6.0 vs. 64.3 ± 8.8 years, *P* < 0.001), we adjusted for age using a linear regression model. After adjustment for age (*β* = 0.144; *P* = 0.135) the CAD_238_ score remained a significant predictor of the Gensini score (*β* =0.418; *P* < 0.001).

To evaluate if factors other than CAD severity influence the CAD_238_ score, we studied control subjects, i.e. those without evidence of CAD (n = 97) (Additional file 1: Table S2). Medication such as beta-blockers, statins, calcium channel blockers, aspirin or angiotensin converting enzyme inhibitors/angiotensin receptor blockers (Additional file 1: Figure S2) as well as cardiovascular risk factors such as hypertension (yes vs no, *n* = 35/58, −0.507 ± 0.344 vs −0.518 ± 0.307, *P* = 0.877), dyslipidaemia (yes vs no, *n* = 42/45, −0.486 vs −0.545, *P* = 0.403), smoking history (yes vs no, *n* = 43/44, −0.542 ± 0.348 vs −0.498 ± 0.300, *P* = 0.510), and diabetes (yes vs no, *n* = 4/93, −0.795 [−0.850; −0.73] vs −0.560 [−0.738; −0.302], *P* = 0.280) did not impact on the CAD_238_ score. There was also no difference in CAD_238_ score between men (*n* = 50) and women (*n* = 47) (−0.480 ± 0.310 vs. −0.568 ± 0.332, *P* = 0.178). In patients without angiographic evidence of CAD, the CAD_238_ score correlated with age as shown in Additional file 1: Figure S3. There was no statistically significant correlation between the CAD_238_ score and carotid intima media thickness in CAD patients (*n* = 73; *ρ* = −0.078, *P* = 0.510) and control subjects (*n* = 70; *r* = 0.011, *P* = 0.926) and thereby no evidence for influences of non-cardiac atherosclerosis on the score.

In 77 % of patients with CAD percutaneous coronary intervention (PCI) was performed prior to urine collection. Considering the correlation between the CAD_238_ and Gensini score we investigated the potential PCI effect on the urine polypeptide pattern. The Gensini score was recalculated by subtraction of stented artery segment (Gensini_corrected_ vs. Gensini score, 6.00 [3.75; 25.25] vs. 35.00 [22.63; 52.38], *P* = 0.001). Using the median of the Gensini_corrected_ score as cut-off the comparison of patients with CAD (*n* = 14) and NCA was repeated (Additional file 1: Figure S4). The difference in CAD_238_ score between patients with CAD and a Gensini_corrected_ score >6 and those with NCA was statistically significant (−0.395 ± 0.386 vs. −0.612 ± 0.269, *P* = 0.036). We used a stepwise linear regression model with CAD_238_ score, age, gender and diabetes status as predictors of CAD to adjust for potential cofounding factors. The resulting model contained only the CAD_238_ score (*β* = 0.208; *P* = 0.053) and gender (*β* = 0.131; *P* = 0.025) and remained statistically significant (*P* = 0.014). Additionally, we correlated the corrected Gensini score with the CAD_238_ score. The result (*ρ* = 0.560, *P* < 0.001) was similar to the correlation between Gensini and CAD_238_ scores.

To estimate a potential value of urine proteomics for the identification of patients with prognostic relevant CAD, we investigated the relationship between the CAD_238_ score and specific angiographic findings in the complete CAD cohort. In accordance with current guidelines [13] prognostically relevant CAD was defined as left main stenosis ≥50 %, three-vessel disease or two-vessel disease including the proximal left anterior descending artery (each with stenosis ≥70 %). The CAD_238_ score difference between prognostically relevant and non-relevant CAD was statistically significant (*n* = 64/32; 0.059 ± 0.442 vs. −0.361 ± 0.416, *P* < 0.001).

## Discussion

The main finding of the present study is the significant association between the CAD_238_ score and extent of CAD. However, contrary to our hypothesis, the CAD_238_ score was not different between patients with chest pain who had CAD and patients with chest pain who had NCA. The retrospective design of our study may account for this apparently negative finding; correcting CAD extent for the treatment effect of PCI resulted in a statistically significant CAD_238_ score difference between patients and controls.

### Urinary proteomics and CAD

Urinary polypeptide patterns have been found to differentiate between patients with and without CAD in a variety of clinical situations such as unstable angina [14] and asymptomatic individuals with high cardiovascular risk [15]. To allow diagnosis of CAD independent of the clinical scenario we previously developed a urinary proteomics-derived pattern consisting of 238 polypeptides [6]. In the present study we evaluated the corresponding CAD_238_ score in patients with or without angiographic CAD.

In contrast to our previous studies in which the CAD_238_ score was developed and validated [6] we have chosen the present study design in order to look into less extreme phenotypes. The study represents a proof of concept study closer to clinical practice [16]. The diagnostic validation of the CAD_238_ score [16] for instance in patients with intermediate cardiovascular risk and stable angina [1, 2] does however require further investigations and further refinement of the CAD specific urine polypeptide pattern.

Scoring systems of coronary angiograms target different qualities. The Syntax score, for instance, evaluates CAD complexity [17]. For this study we chose the Gensini Score [7] in order to quantify CAD extent/severity. In the past the Gensini score has been associated with inflammation [18], systemic atherosclerosis [19], peripheral vascular disease [20], renal impairment [21] and the chromosome 9p21 risk locus [22] emphasising its value in CAD assessment.

Gensini scores in patients with CAD and stable angina in the present study were lower than those in patients awaiting CABG in our previous biomarker validation study, confirming the expected less pronounced coronary atherosclerosis in the present cohort [6]. Also, the majority of patients with CAD and stable angina underwent PCI including stent insertion prior to urine collection. Intervention leads to extensive arterial wall remodeling and neointima formation [23, 24]. The altered physiology of the stented artery segment is therefore expected to impact on the CAD_238_ score. We therefore accounted for the reduction of atherosclerotic wall segments in contact with the blood stream after PCI. The difference in CAD_238_ score between control subjects and patients with remaining significant coronary artery atheroma after PCI, represented by a Gensini score >6, was statistically significant. The intervention probably contributed to a “healthier” CAD_238_ score in patients with stable angina and CAD than in those scheduled for surgical coronary revascularization who were previously studied.

We found, however, a strong correlation between the Gensini score and the CAD_238_ score. The Gensini score was designed to reflect on the number of diseased coronary arteries, their importance regarding dependent myocardial mass and the extent of arterial narrowing [7]. The score therefore provides a measure of CAD extent. In extensive CAD the surface area of plaque will be larger. Therefore more atherosclerosis related peptides will enter the blood stream and can be subsequently detected in the urine.

In our cohort carotid intima-media thickness and the CAD_238_ score were not correlated. As carotid intima-media thickness is a marker of early atherosclerosis, the finding suggests the independence of the CAD_238_ score from non-cardiac atherosclerosis.

Next to the diagnosis of significant CAD in stable angina patients, the identification of prognostic relevant CAD holds clinical value. Several predictors of prognostically relevant CAD are known [25]. Considering the significant CAD_238_ score difference between prognostically relevant and non-relevant CAD in our cohort, urine proteomics has also potential to identify patients with such disease and to direct therapies to high-risk patients.

### Collagen turnover assessment by urinary proteomics

Most peptides in the CAD_238_ panel were collagen fragments [6]. As the collagen content in artery walls increases with age [26], leading to increased arterial wall stiffness, this might explain the score’s correlation with age. Altered degradation of Type 1 and 3 collagens in atheromatous plaque [27] might also explain the predominance of collagen fragments, especially since plaque burden increases with age [28]. In older subjects the collagen concentration in atheromatous plaque increases in areas close to the arterial lumen [29] and is therefore in closer contact with the blood stream.

Synthesis or degradation of collagens is slow in a healthy arterial system [30], which may imply decreased levels of collagen fragments in the blood stream of healthy individuals. In atheromatous plaque the main component of the fibrous cap, the region in contact with the circulating blood, consists of types 1 and 3 collagens [31]. Atherosclerosis causes an increased synthesis and degradation of many matrix components [32], and the CAD_238_ score derived from urinary proteomics may represent a measure of collagen turnover and thereby represent a biomarker of cardiovascular disease.

Significant CAD not only affects the coronary vessel walls but also the myocardium by transient or permanent ischemia; for example in myocardium with exercise induced perfusion deficits or hibernating myocardium. Considering the statistically significant differences in CAD_238_ score after correction of the Gensini score as well as the high CAD_238_ score in prognostically relevant CAD, myocardial ischemia on its own or in addition to cardiac atherosclerosis could potentially contribute to the urinary proteome in patients with CAD. Transient [33] or permanent [34] inadequate myocardial perfusion leads to localized inflammation and increased interstitial fibrosis. As myocardial fibrosis is characterized by accumulation of collagen types 1 and 3 within the intercellular space [35], this quantity change implies increased collagen degradation and hence a rise in collagen fragments.

### Study limitations

The main finding of the present study, i.e. the relationship between CAD_238_ score and CAD extent, is at the same time the major limitation of urine proteomics as a diagnostic tool for CAD. Indeed, our present findings suggest that the CAD-specific urinary polypeptide pattern is related to CAD extent and it should of course be noted that normal coronary angiography results do not exclude early forms of coronary atherosclerosis. The polypeptide pattern could in addition to or instead of coronary atherosclerosis identify flow limiting CAD with consecutive myocardial fibrosis. Therefore, additional investigations are required to determine the origin of the CAD_238_ score.

A further limitation of the present study is its retrospective design. Urine samples were collected after coronary angiography, which included PCI in 77 % of the cases. It is therefore possible that the CAD_238_ score could differentiate between patients with stable angina with and without CAD in a prospective study if the samples had been taken prior to PCI.

## Conclusions

We have previously shown that polypeptide patterns derived form urine proteomics provides good diagnostic accuracy for severe CAD. The CAD_238_ score is, however, of limited diagnostic value in patients with stable angina-like chest pain who have less extensive CAD. Furthermore, the CAD_238_ score reflects on CAD extent suggesting that the score has potential for prognostication but is less useful for diagnosis in patients with stable angina if not complemented by other diagnostic tests. However, our data support the association of urinary peptides with CAD and provide a rationale for further development of diagnostic proteomic biomarkers.

### Ethics approval and consent to participate

All participants gave written informed consent. The study was approved by the West of Scotland Research Ethics Committee and is in keeping with the principles of the Declaration of Helsinki.

### Consent for publication

Not applicable.

### Availability of data and materials

Details on the peptides that determine the CAD_238_ score have been published previously [6]. Further data are available on request from the corresponding author.

## Additional file

Additional file 1: Table S1. Cohort characteristics of patients with severe CAD planned to undergo CABG surgery and age matched healthy volunteers. Table S2. Cohort characteristics of combined patients with CAD and controls (NCA or healthy volunteers). Figure S1. Flowchart of the DiCADu study design and recruitment process. CAD, coronary artery disease; NCA, normal coronary arteries. Figure S2. Effect of drug treatment on the CAD_238_ score. For comparisons Student’s *t*-test was used. ACEI, angiotensin-converting enzyme inhibitor; ARB, angiotensin receptor blocker; CCB, calcium channel blocker; BB, beta-blocker. Figure S3. Correlation between age and CAD_238_ score in control subjects. The Pearson correlation coefficient and the corresponding *P*-value are shown. Figure S4. Comparison of CAD_238_ score between patients with NCA and CAD corrected for remaining coronary atherosclerosis after percutaneous coronary intervention. Lines represent the median. NCA, normal coronary arteries. (DOC 1181 kb)

## Abbreviations

ACEI: angiotensin-converting enzym
ACR: albumin-creatinine ratio
ARB: angiotensin receptor blocker
BMI: body mass index
CABG: coronary artery bypass graft
CAD: coronary artery diseasse
DBP: diastolic blood pressure
HDL: high-density lipoprotein
ICR: interquartile range
IMT: intima-media thickness
LDL: low-density lipoprotein
NCA: normal coronary arteries
PCI: percutaneous coronary intervention
SBP: systolic blood pressure
