# Supplementary material for: Urine proteomics in the diagnosis of stable angina
Source: BMC Cardiovasc Disord. 2016 Apr 19;16:70. doi: 10.1186/s12872-016-0246-y (PMC4837614; doi:10.1186/s12872-016-0246-y)

**Urine proteomics in the diagnosis of stable angina**

Ulf Neisius ^a^, PhD, Thomas Koeck ^b^, PhD, Harald Mischak ^a,b^, PhD, Sabrina H Rossi ^a^,, MB ChB, Erin Olson ^a^, MSc, David M Carty ^a^, PhD, Jane A Dymott ^a^, MD, Anna F Dominiczak ^a^, MD, Colin Berry ^a,c^, PhD, Keith G Oldroyd ^a,c^, MD, Christian Delles ^a^, MD

a BHF Glasgow Cardiovascular Research Centre, Institute of Cardiovascular and Medical Sciences, University of Glasgow, 126 University Place, Glasgow G12 8TA, United Kingdom

b mosaiques diagnostics GmbH, Mellendorfer Strasse 7-9, 30625 Hannover, Germany

c Golden Jubilee National Hospital, Agamemnon Street, Clydebank G81 4DY, United Kingdom

**Additional file**

Table S1. Cohort characteristics of patients with severe CAD planned to undergo CABG surgery and age matched healthy volunteers.

|  | CABG, n=66 | Healthy Volunteers, n=67 | *P*-value |
| --- | --- | --- | --- |
| CAD_238_ score | 0.110±0.401 | -0.483±0.338 | <0.001 |
| ACR (all > detection limit) | 1.2 [0.8; 2.0] | 0.9 [0.7; 1.6] | 0.191 |
| Age, years | 64.3±8.8 | 61.9±8.4 | 0.103 |
| Sex, m/f | 51/15 | 41/26 | 0.023 |
| BMI, kg/m2 | 29.1±6.2 | 26.0±3.5 | <0.001 |
| SBP, mmHg | 139±25 | 138±19 | 0.698s |
| DBP, mmHg | 78±12 | 82±11 | 0.075 |
| Heart rate, /min | 64±12 | 68±13 | 0.039 |
| Total cholesterol, mmol/l | 4.1 [3.5; 4.8] | 5.8 [5.1; 6.5] | <0.001 |
| LDL-cholesterol, mmol/l | 2.1 [1.5; 2.4] | 3.4 [2.8; 4.5] | <0.001 |
| HDL-cholesterol, mmol/l | 1.2 [1.0; 1.3] | 1.5 [1.2; 1.8] | <0.001 |
| Trilycerides, mmol/l | 1.8 [1.4; 2.6] | 1.3 [1.0; 2.0] | <0.001 |
| Hypertension History, % | 62 | 29 | 0.001 |
| CAD Family History, % | 26 | 34 | 0.450 |
| Diabetes History, % | 23 | 0 | <0.001 |
| Active smoking, % | 10 | 7 | 0.744 |
| Statin, % | 88 | 12 | <0.001 |
| Aspirin, % | 85 | 13 | <0.001 |
| Beta-blocker, % | 83 | 7 | <0.001 |
| ACEI/ARB, % | 59 | 7 | <0.001 |
| Gensini-Score | 77 [56; 109] | - | - |
| Carotid IMT, mm | 0.803±0.167 * | 0.689±0.152 # | 0.002 |

Both cohorts have been previously described by Delles et al. [6]. Data are given as mean±SD or median [ICR] as appropriate. *P*-values are from Student’s t-test, Mann-Whitney U-test, Chi-square test or Fisher’s exact test where appropriate. ACEI, angiotensin-converting enzyme inhibitor; ARB, angiotensin receptor blocker; CAD, coronary artery disease; IMT, intima-media thickness; NCA, normal coronary arteries; HDL, high-density lipoprotein; LDL, low-density lipoprotein; * n=44; # n=42.

Table S2. Cohort characteristics of combined patients with CAD and controls (NCA or healthy volunteers).

|  | Cases, n=96 | Controls, n=97 | P-value |
| --- | --- | --- | --- |
| CAD_238_ score | -0.077±0.472 | -0.523±0.322 | <0.001 |
| ACR (all > detection limit) | 1.1 [0.8; 1.9] | 1.1 [0.7; 1.8] | 0.558 |
| Age, years | 61±9 | 60±8 | 0.244 |
| Sex, m/f | 67/29 | 50/47 | 0.009 |
| BMI, kg/m2 | 28.3 [25.3; 31.1] | 26.1 [23.4; 30.0] | 0.003 |
| SBP, mmHg | 139±23 | 138±19 | 0.741 |
| DBP, mmHg | 78±11 | 81±10 | 0.031 |
| Heart rate, /min | 60 [54; 67] | 63 [56; 76] | 0.032 |
| Total cholesterol, mmol/l | 4.3±0.9 | 5.5±1.2 | 0.002 |
| LDL-cholesterol, mmol/l | 2.1 [1.7; 2.7] | 3.3 [2.4; 3.9] | <0.001 |
| HDL-cholesterol, mmol/l | 1.2 [1.0; 1.4] | 1.4 [1.2; 1.7] | <0.001 |
| Triglycerides, mmol/l | 1.8 [1.3; 2.5] | 1.4 [1.0; 2.2] | 0.031 |
| Hypertension History, % | 66 | 40 | <0.001 |
| CAD Family History, % | 41 | 45 | 0.563 |
| Diabetes History, % | 22 | 4 | <0.001 |
| Active smoking, % | 13 | 10 | 0.497 |
| Statin, % | 87 | 26 | <0.001 |
| Aspirin, % | 87 | 20 | <0.001 |
| Beta-blocker, % | 83 | 13 | <0.001 |
| ACEI/ARB, % | 53 | 16 | <0.001 |
| Gensini-Score | 61 [42; 100] | - | - |
| Corrected Gensini-Score^‡^ | 57 [17; 97] |  |  |
| Carotid IMT, mm | 0.738 [0.675; 0.873] * | 0.715±0.137 # | 0.018 |

Data are given as mean±SD or median±ICR as appropriate. *P*-values are from Student’s t-test, Mann-Whitney U-test, Chi-square test or Fisher’s exact test where appropriate. ACEI, angiotensin-converting enzyme inhibitor; ARB, angiotensin receptor blocker; CAD, coronary artery disease; IMT, intima-media thickness; NCA, normal coronary arteries; HDL, high-density lipoprotein; LDL, low-density lipoprotein; ‡ Gensini-score after percutanous intervention; * n=73; # n=70.

SUPPORTING FIGURE LEGENDS

Figure S1. Flowchart of the DiCADu study design and recruitment process. CAD, coronary artery disease; NCA, normal coronary arteries.

Figure S2. Effect of drug treatment on the CAD_238_ score. For comparisons Student's t-test was used. ACEI, angiotensin-converting enzyme inhibitor; ARB, angiotensin receptor blocker; CCB, calcium channel blocker; BB, beta-blocker.

Figure S3. Correlation between age and CAD_238_ score in control subjects. The Pearson correlation coefficient and the corresponding P-value are shown.

Figure S4. Comparison of CAD_238_ score between patients with NCA and CAD corrected for remaining coronary atherosclerosis after percutaneous coronary intervention. Lines represent the median. NCA, normal coronary arteries.

Figure S1


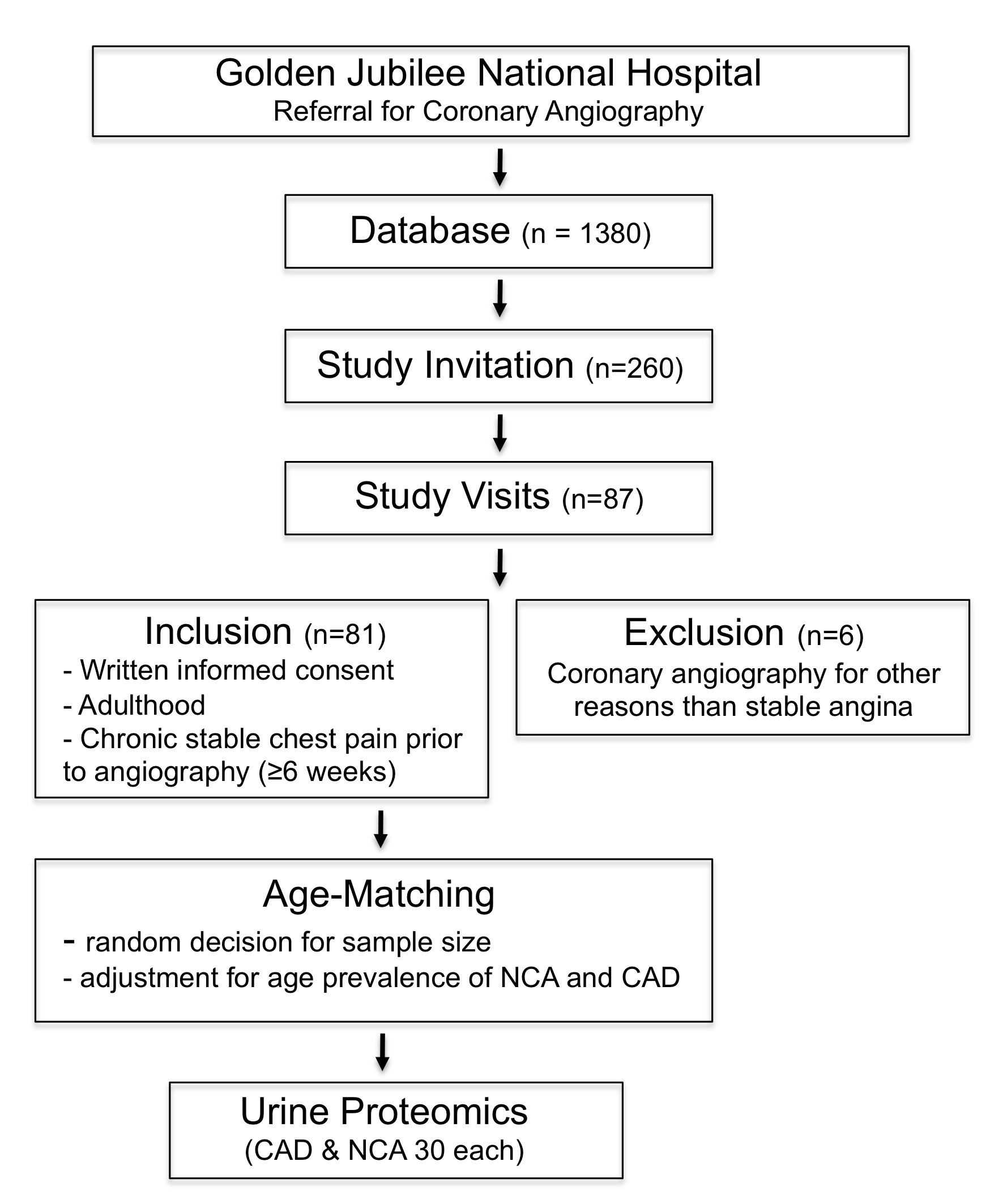


Figure S2


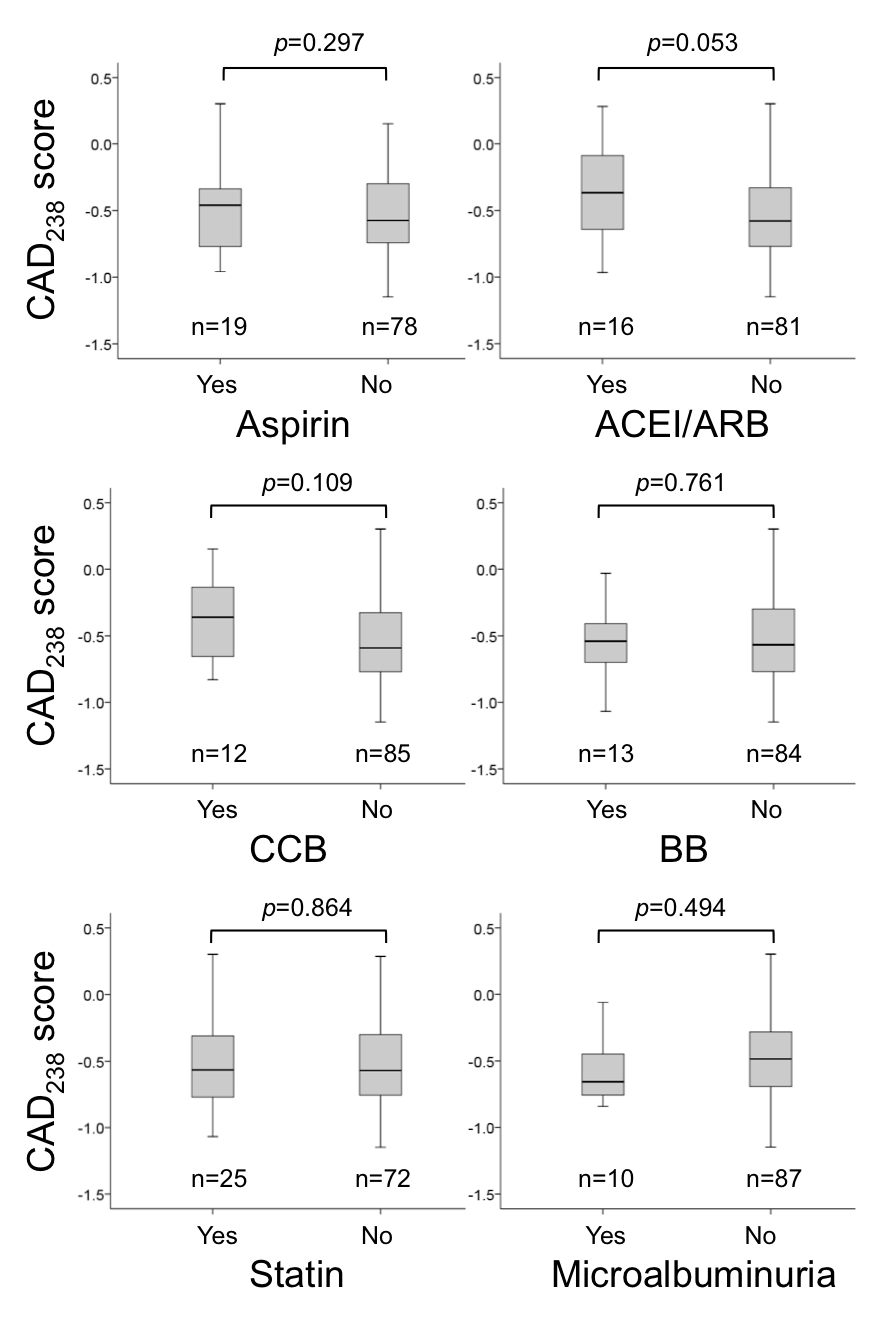


Figure S3.


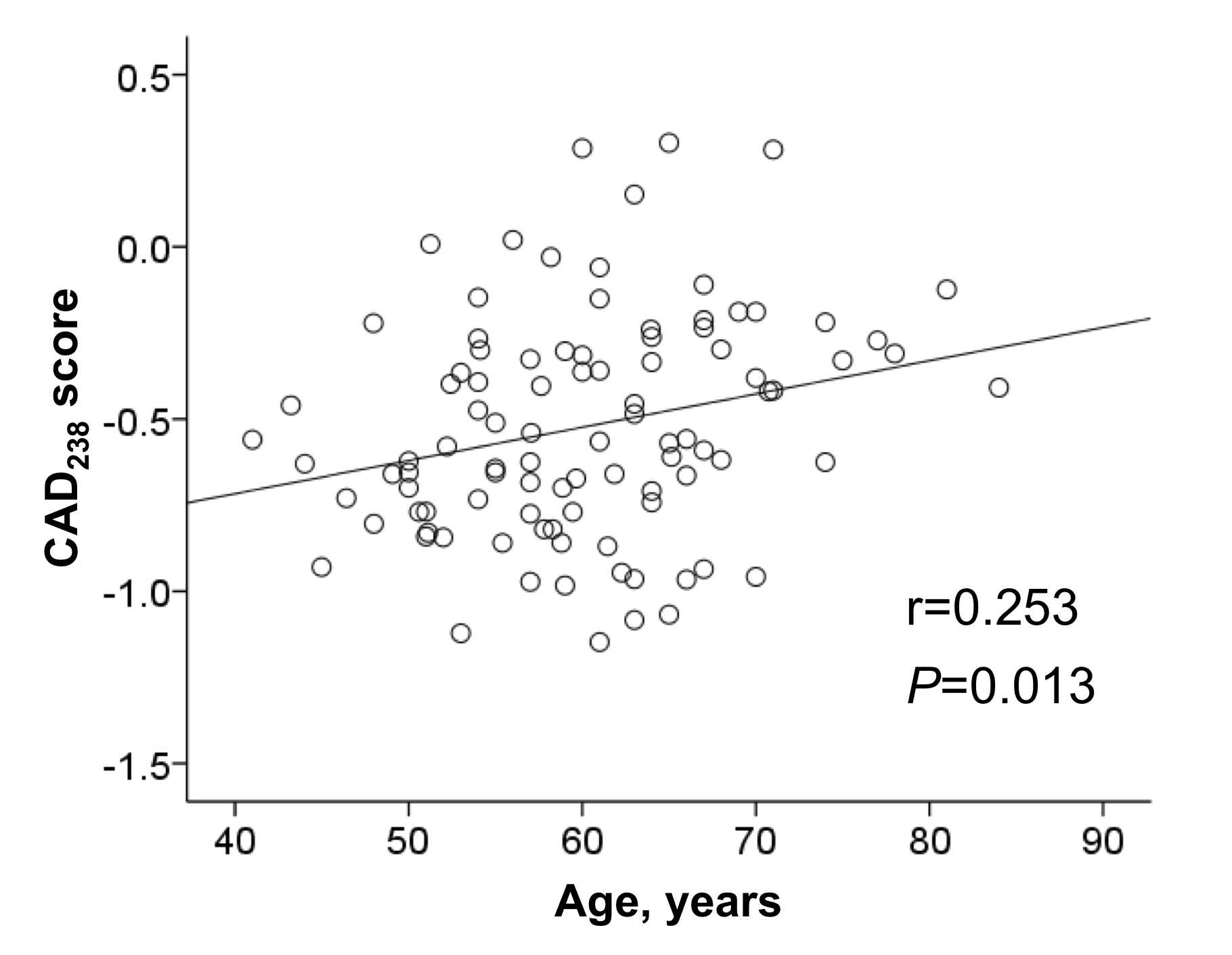


Figure S4.


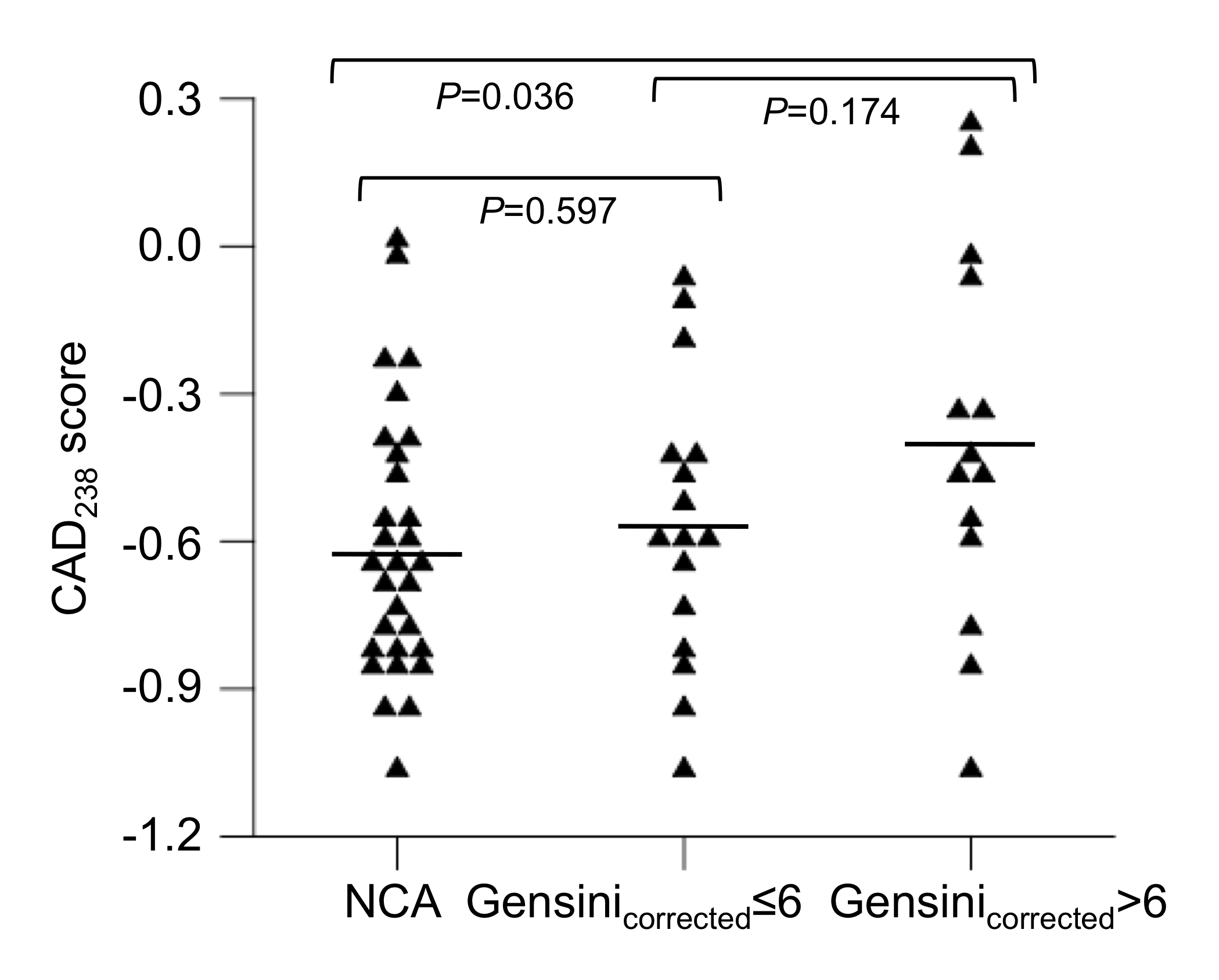

Supplement: Additional file 1: Table S1. — Cohort characteristics of patients with severe CAD planned to undergo CABG surgery and age matched healthy volunteers. Table S2. Cohort characteristics of combined patients with CAD and controls (NCA or healthy volunteers). Figure S1. Flowchart of the DiCADu study design and recruitment process. CAD, coronary artery disease; NCA, normal coronary arteries. Figure S2. Effect of drug treatment on the CAD238 score. For comparisons Student’s t-test was used. ACEI, angiotensin-converting enzyme inhibitor; ARB, angiotensin receptor blocker; CCB, calcium channel blocker; BB, beta-blocker. Figure S3. Correlation between age and CAD238 score in control subjects. The Pearson correlation coefficient and the corresponding P-value are shown. Figure S4. Comparison of CAD238 score between patients with NCA and CAD corrected for remaining coronary atherosclerosis after percutaneous coronary intervention. Lines represent the median. NCA, normal coronary arteries. (DOC 1181 kb) [file 12872_2016_246_MOESM1_ESM.doc]
